# Induction of J Aggregate-like Optical Transitions in Dihydroxyquinone by Coordination with Al(III)

**DOI:** 10.1021/acs.jpca.5c06958

**Published:** 2026-01-29

**Authors:** José Roberto Granado Neto, Antonio Gustavo Sampaio de Oliveira-Filho, Marcelo Henrique Gehlen

**Affiliations:** Department of Physical Chemistry, Institute of Chemistry of São Carlos, 28133 University of São Paulo, São Carlos, São Paulo 13566-590, Brazil

## Abstract

The metal-complex formation of 1,4-dihydroxyanthraquinone (quinizarin, QNZ) and 6,11-dihydroxy-5,12-naphthacenedione (DHN) with Al­(III) ions is investigated by stationary and time-resolved emission spectroscopy combined with quantum chemical calculations of optical properties. UV–vis and fluorescence spectra revealed small red-shifts of 200 and 60 meV for the QNZ and DHN metal complexes, respectively. The fluorescence quantum yield increases from 0.08 to 0.23 for QNZ, while for DHN it changes from 0.24 to 0.79 upon complexation, suggesting the presence of J-aggregate-like exciton coupling within the coordination structure. The average fluorescence lifetime of QNZ varies from 0.65 ns of the free ligand to 2.77 ns, and in the case of DHN it goes from 1.57 to 2.61 ns after Al­(III) complexation. These results are consistent with formation of a more rigid molecular structure which effectively decreases the nonradiative rate constant. Confocal fluorescence microscopy images of Al­(III) complexes adsorbed into the μmZeolite L structure gave similar red-shifted J type emission. Density functional theory, at the B3LYP/def2-TZVP level of theory, and the analysis of the electronic transition dipole moment, calculated with TDDFT at the CAM-B3LYP/def2-TZVP level, supports a near head-to-tail chromophore arrangement containing two metal centers coordinated with two chromophores. The Al­(III)_2_DHN_2_ complex exhibits the stronger transition dipole coupling and a more pronounced J-type character when compared with Al­(III)_2_QNZ_2_ complex. The radiative rate constant of Al­(III)_2_DHN_2_ is twice that of the single DHN chromophore.

## Graphical abstract

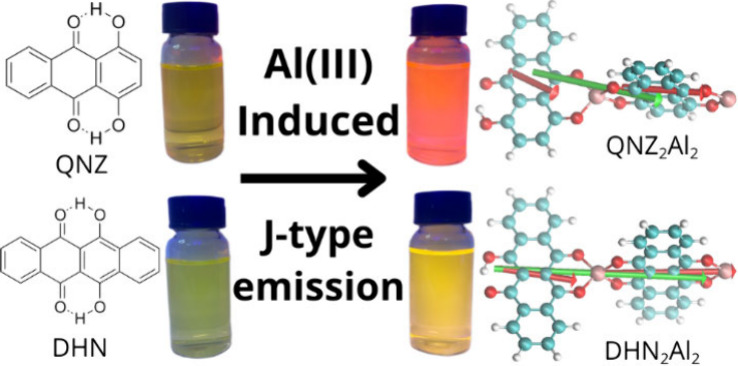

## Introduction

Dihydroxyquinones (DHQ) are organic molecules known for their prominent photophysical, photocatalytic, redox, and coordination properties allowing applications in many industrial fields.^1–6^ These compounds are currently used as anticancer, antibiotics besides other medicinal uses.^7,8^ The structural differences among the isomeric hydroxyl substitutions significantly impact their electronic and optical behavior, making them valuable models for structure–property relationship studies.^9^ In addition, such compounds may present intramolecular resonance assisted hydrogen bonding (RAHB), which extends and lowers the energy of the conjugated π-system, and therefore modulates their molecular properties.^10^ 1,4-Dihydroxyanthraquinone (quinizarin, QNZ) and 6,11-dihydroxy-5,12-naphthacenedione (DHN) have also demonstrated a strong metal chelation capacity.^11–15^

It has been proposed that quinizarin forms polymeric complexes with Al­(III) and other metals in a 1:1 metal/QNZ ratio in both aqueous and nonaqueous solutions.^11,12,16,17^ These complexes display strong fluorescence, red-shifted electronic absorption, a high molar absorption coefficient, and a small Stokes shift. These characteristics strongly correlate with the formation of extended molecular aggregates.^18–20^ The coordination of organic chromophores with Al­(III) has also been investigated owing to their high fluorescence quantum yields attained that paved the way to their applications in fluorescence sensors and OLED materials.^21–25^

A well-known type of organic aggregate is the classical J-aggregate, which forms through head-to-tail packing of molecules with nearly collinear transition dipole moments.^26–28^ They are characterized by a red-shifted absorption band, increased molar absorption coefficients, superradiance, and faster fluorescence decay.^29–34^ Recent studies have also shown that metal ions can directly promote J-type aggregation in organic chromophores. In particular, Zn­(II) has been reported to induce red-shifted, strongly emissive J-aggregates, illustrating how metal coordination can organize chromophores into exciton-coupled assemblies.^35^

In the present contribution, we seek to elucidate the fundamental nature of the intrinsic spectroscopic properties of Al­(III) complexes with quinizarin (QNZ) and DHN (molecular structures are given in Figure 1). Absorption and emission measurements and fluorescence decay analysis in solution as well as when the complexes are loaded in microzeolite L, combined with DFT quantum chemical calculations, pointed out the emergence of optical transitions of J-type character in these metal-complex aggregates.

**Figure 1:**
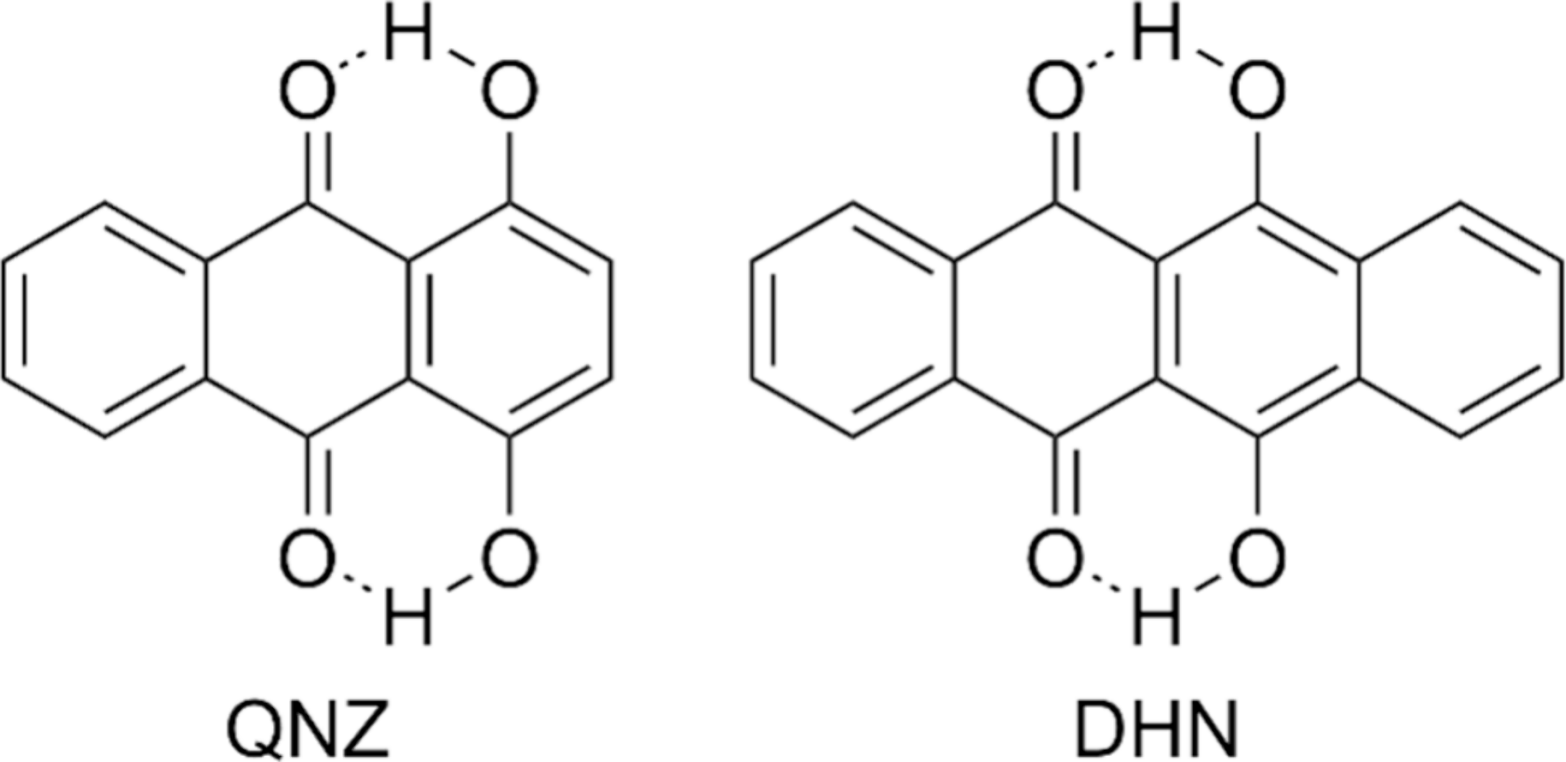
Molecular structure of 1,4-dihydroxyanthraquinone (quinizarin, QNZ), 6,11-dihydroxy-5,12-naphthacenedione (DHN) with the RAHB effect.

## Experimental and Theoretical Methods

Ethanol, 1,4-dihydroxyanthraquinone and 6,11-dihydroxy-5,12-naphthacenedione were from Sigma-Aldrich, and Al­(NO)_3_·9H_2_O) was purchased from Fluka Co. The initial concentrations of QNZ and DHN in ethanol were 5 × 10^–5^ mol/L, while the concentration of Al­(III) in the system was increased by adding small amounts of Al­(NO)_3_·9H_2_O concentrated ethanol solution, getting to molar equivalents ranging from 0.2 to 20 at room temperature; all solutions rested at least 12 h before analysis for stabilization. To load the complexes into the μmZeolite-L particles,^36^ the μmZeolite-L were dispersed in 1 mL of either QNZ or DHN solution containing 20 mol equiv of Al­(III) and left to equilibrate for 2 h. After incubation, the particles were washed three times with ethanol to remove nonincorporated chromophores. The resulting chromophore-loaded zeolites were then deposited onto clean glass coverslips by spin-coating (Ossila spin coater), starting at 1000 rpm and ramping to 4500 rpm, to obtain homogeneous thin films.

### Spectroscopic and Fluorescence Microscopy Measurements

UV–vis and fluorescence spectra of stabilized samples at 20 °C were recorded on a Shimadzu UV-1800 spectrophotometer and a Hitachi F-4500 spectrofluorometer, respectively. Fluorescence quantum yields (Φ_*f*_) were calculated relative to rhodamine B in ethanol (Φ_std_ = 0.49)^37^ according to eq 1:

1 $$\Phi_{f}=\frac{I_{f}}{I_{\mathrm{std}}}\frac{A_{\mathrm{std}}}{A_{f}}\Phi_{\mathrm{std}}$$

where the subscripts f and std represent sample and standard, respectively, *I* is the integral area of fluorescence emission, and *A* is the absorbance at very low absorption, and the measurements were done in a special 2 × 2 mm^2^ path length quartz cuvette (Hellma GmbH & Co.) as the solutions used were slightly concentrated. Calculations of radiative decay rate constant (*k*_f_) and nonradiative decay rate constant (*k*_nr_) were done by using eqs 2 and 3, respectively:

2 $$k_{f}=\frac{\Phi_{f}}{\tau }$$

3 $$k_{\mathrm{nr}}=\frac{1-\Phi_{f}}{\tau }$$

where Φ_f_ is fluorescence quantum yield and τ is fluorescence lifetime.

Infrared spectra from the pure and labeled μmZeolite-L were obtained in a Tensor 27 (Bruker, USA) under the attenuated total reflection (ATR) mode.

Confocal fluorescence images were measured in an Olympus IX71 microscope equipped with a digital piezoelectric controller and stage (PI, E-710.3CD and P-517.3CD) for precise nanometric scanning.^38^ A laser diode (QuixX 473-100 PS, Omicron, Germany), operating at 2 MHz pulse frequency, provided excitation pulses of about 100 ps at 473 nm. The excitation light was circularly polarized using λ/2 and λ/4 waveplates (models AHWP05M-600 and AQWP05M-600 from Thorlabs) and focused onto the sample with a 100X Olympus UPLFLN objective. The emitted signal was isolated from the excitation laser using a dichroic cube (Chroma, z470 rd) and a notch filter (Chroma, ZET473NF). A PerkinElmer SPCM-AQR-14 avalanche photodiode point detector, aligned with a 50 mm pinhole, was used to count the emission photons. Logic signals were acquired by a counter-timer card (NI 6601) and transferred to a PC computer. A scanning control program written in C# was used to generate 2D plots using false-color mapping to achieve the best contrast enhancement based on fluorescence intensity differences.

A single-photon timing technique with a Becker & Hickl 140 counting board measured fluorescence decays. The start laser pulses were triggered by a Pico-Quant TDA 200 photodiode. Time-resolved decays were fitted using the FAST software (Edinburgh Instruments). The measured instrument response function (IRF) was obtained from the backscattering of a clean coverslip without ND filter in the confocal line, and it was used for reconvolution with multiexponential decay models to extract fluorescence decay times.

Lastly, a spectrometer (Maya 2000 Pro, Ocean Optics) connected to the right-side port of the Olympus IX71 microscope with an optical fiber was used to obtain sample emission spectra.

### Theoretical Study (DFT and TD-DFT)

Density Functional Theory (DFT) calculations were performed with ORCA 6.1,^39^ using the def2-TZVP as basis set^40,41^ and the B3LYP functional^42,43^ for geometry optimizations and CAM-B3LYP^44^ for TDDFT calculations. The vector decomposition analysis of the transition dipole moment was performed using Multiwfn^45^ and visualized using VMD.^46^ To confirm the nature of the optimized geometries as true minima on the potential energy surface, vibrational frequency analyses were carried out. The absence of imaginary frequencies confirmed that all of the optimized structures were a local minimum.

## Results and Discussion

### Spectroscopy in Solution

The UV–vis spectra of QNZ in ethanol upon the addition of Al­(III) ions are illustrated in Figure 2a. QNZ solution, which appears as yellow-orange solution, displays a broad absorption band with maximum at 481 nm. With successive additions of Al­(III) ions, a distinct color change is observed, progressing through purple and culminating in a stable brightened pink color at 10 or more mol equiv of Al­(III)/QNZ (see Supporting Information (SI) for solution picture and CIE diagram). Its absorption band is characterized by new maxima at 488, 520, and 560 nm. The fluorescence spectrum of quinizarin (QNZ) also changes significantly upon addition of Al­(III) ions, and the emission spectra are reported in Figure 2b. The emission band of QNZ with maxima at 535 and 562 nm is initially quenched upon addition of Al­(III). However, at high concentration of Al­(III), a strong red-shifted emission band with maximum at 572 and 608 nm appears. Additionally, a significant change in the S_0_ → S_1_ energy values (E_0–0_) and Stokes shift is observed upon complexation. For instance, for QNZ in ethanol, the E_0–0_ is 2.39 eV (518 nm) with a Stokes shift of 54 nm, but at high Al­(III), the E_0–0_ decreases to 2.19 eV (565 nm) with a small Stokes shift of only 12 nm.

**Figure 2:**
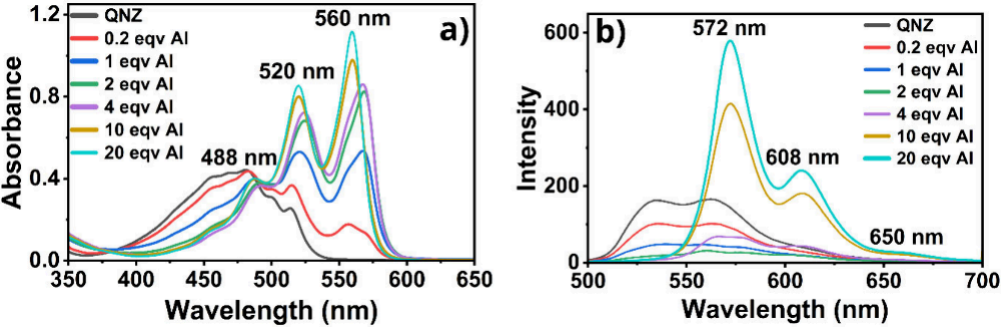
a) UV–vis and b) fluorescence spectra for quinizarin and QNZ/Al­(III) (λ_exc_ = 483 nm).

The changes in the electronic absorption spectrum of 6,11-dihydroxy-5,12-naphthacenedione (DHN) upon the addition of Al­(III) ions are given in Figure 3a. DHN solution in ethanol, which appears yellow-orange, has absorption maxima at 453, 481, and 515 nm, while with successive additions of Al­(III) ions, it changes to a strong structured band with new absorption maxima at 469, 501, and 538 nm, making the solution color bright orange (see SI for solution picture and CIE diagram). The fluorescence spectrum of DHN undergoes substantial changes in the presence of Al­(III) ions, as shown in Figure 3b. The pure DHN exhibits an emission band with maxima at 527 and 560 nm, and a shoulder at 606 nm. Upon complexation with Al­(III) at high concentration, a new strong emission band appears with maxima at 551 and 583 nm, and a shoulder at 628 nm. Furthermore, E_0–0_ shifts from 2.34 eV (529 nm) in DHN to 2.28 eV (543 nm) at 20 mol equiv of Al­(III), and the Stokes shift remains small at 12 nm.

**Figure 3:**
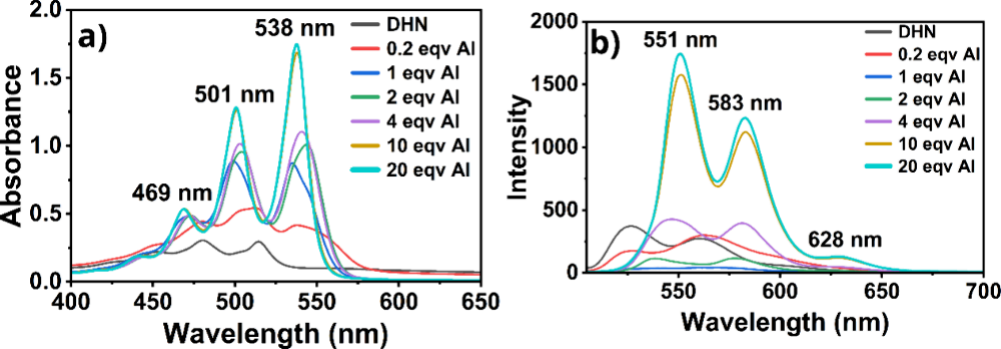
a) UV–vis and b) fluorescence spectra for DHN and DHN/Al­(III) (λ_exc_ = 497 nm).

The fluorescence decay of QNZ in the presence of varying molar equivalents of Al­(III) is reported in Figure 4a. Pure QNZ exhibits a short average fluorescence decay time of 0.65 ns. Upon complexation with Al­(III), the average decay time significantly increases, reaching 2.77 ns at a 20:1 Al­(III):QNZ ratio. At this higher concentration, the decay is weighted equally by two distinct time constants: 1.25 and 3.34 ns. This change in decay kinetics suggests that the interaction with Al­(III) seems to disrupt resonance-assisted hydrogen bonding (RAHB) by exchanging H^+^ with the Al­(III) anion.

**Figure 4:**
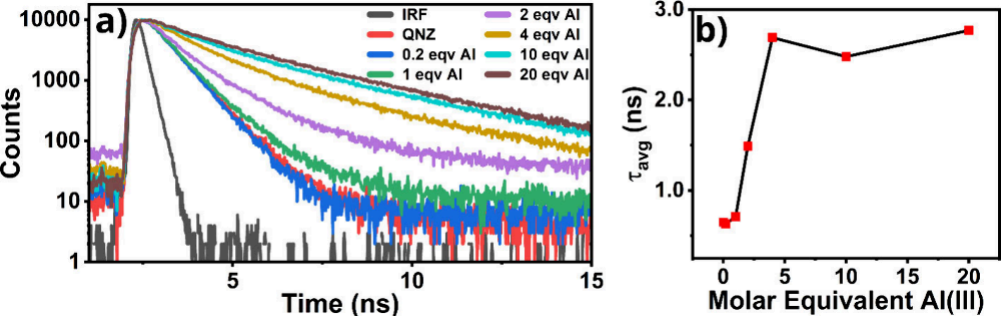
a) Fluorescence decay of quinizarin and QNZ/Al. b) Average fluorescence decay time as a function of Al (III) molar equivalent for QNZ/Al.

Additionally, the fluorescence decay of DHN in the presence of varying molar equivalents of Al­(III) is given in Figure 5a. Pure DHN exhibits a double-exponential decay with an average fluorescence decay time of 1.57 ns, primarily influenced by its longer decay time of 1.61 ns. Upon complexation with Al­(III), at both 10:1 and 20:1 Al­(III):DHN ratios, the decay becomes exponential, with a single lifetime of 2.61 ns.

**Figure 5:**
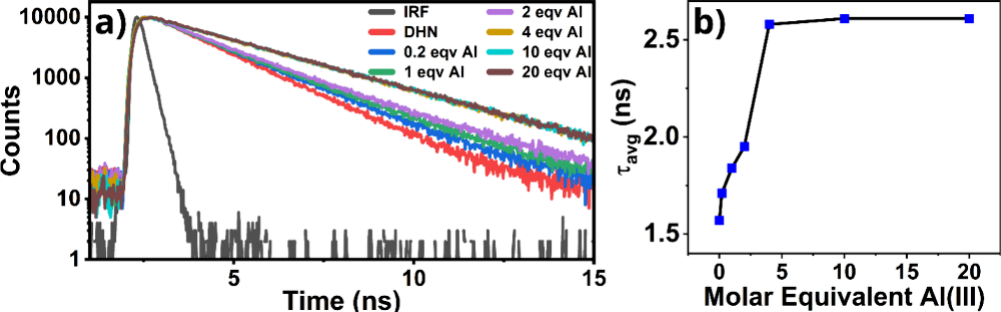
a) Fluorescence decay of quinizarin and DHN/Al. b) Average fluorescence decay time as a function of Al­(III) molar equivalent for DHN/Al.

The average decay time of the fluorescence increases in both systems, as shown in Figure 4b and 5b. Also, the values get a saturation after a molar equivalent of Al­(III) of about 5 with respect to the initial concentration of QNZ and DHN. This pronounced increase in fluorescence lifetime departs from the canonical J-aggregate exciton-coupling behavior, although analogous exceptions have been reported for other aggregate systems.^47^

The calculated fluorescence quantum yields (relative to rhodamine B in ethanol with 500 nm excitation) increase substantially upon complexation with Al­(III). QNZ increases from 0.08 to 0.23, while the DHN varies from 0.24 to 0.79. Analysis of the rate constants pointed out that in the QNZ–Al­(III) complex, the radiative rate constant remains in the same range, and the dominant effect for quantum yield enhancement comes from the reduction in the nonradiative rate constant from 1.45 to 0.28 ns^–1^. For DHN–Al­(III), the increase in *k*_f_ from 0.15 to 0.30 ns^–1^ and the decrease in *k*_nr_ from 0.48 to 0.08 ns^–1^ both contribute to quantum yield enhancement. These results are summarized in Table 1.

**Table 1:** Quantum yield (Φ_*f*_), Average Fluorescence Lifetime (τ_avg_), Radiative (*k*_*f*_) and Non-radiative (*k*_*nr*_) Rates for QNZ, DHN and in Al­(III) solutions

Sample	Φ_*f*_	τ_avg_ (ns)	*k*_*f*_ (ns^–1^)	*k*_nr_ (ns^–1^)
QNZ	0.08	0.65	0.12	1.45
QNZ:Al(III) (1:20)	0.23	2.77	0.08	0.28
DHN	0.24	1.57	0.15	0.,48
DHN:Al(III) (1:20)	0.79	2.61	0.3	0.08

In dilute solution, the aggregation process upon addition of Al (III) may be represented by eq 4 with chainlike formation that under high concentration of Al will favor the binary form Al_2_DHQ_2_ (DHQ: QNZ or DHN) due to charge restriction and a lower amount of initial concentration of the DHQ.

4 $$\mathrm{DHQ}\overset{{\mathrm{Al}}^{3+}}{⇄}{\mathrm{AlDHQ}}^{+1}\overset{{\mathrm{Al}}^{3+}}{⇄}{\mathrm{Al}}_{2}{\mathrm{DHQ}}^{+4}\overset{\mathrm{DHQ}}{⇄}{\mathrm{Al}}_{2}{\mathrm{DHQ}}_{2}^{3+}$$

The aggregation kinetics in terms of the change of absorption as a function of time were further analyzed using a pseudo-second-order model, which provided an excellent fit to the experimental data (Figure 6). The model indicates that dynamics of the aggregation process is governed by the availability of active coordination sites, consistent with a stepwise complexation mechanism given in eq 1. The rate constants and respective aggregation half-times obtained highlight distinct kinetic profiles for QNZ (*t*_1/2_ = 7.9 min) and DHN (*t*_1/2_ = 18.5 min), reflecting their different coordination strengths with Al­(III).

**Figure 6:**
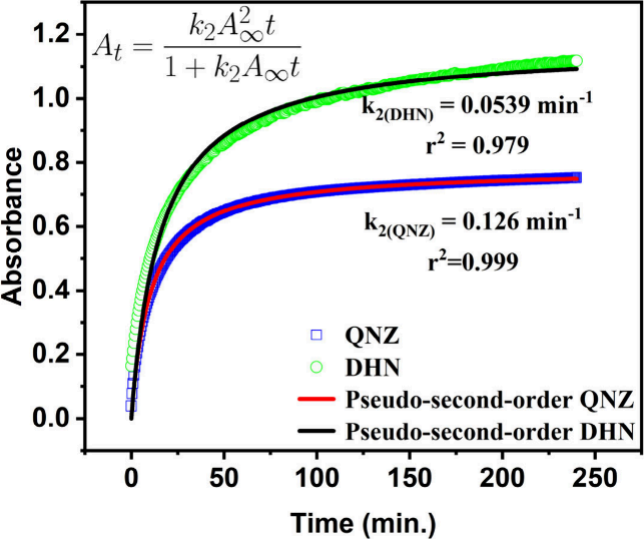
Pseudo-second-order kinetic model describing the aggregation of QNZ and DHN with Al^3+^, by absorbance vs time measurement. Experimental data are represented by symbols and the corresponding model fits by solid lines.

### Fluorescent in Labeling μmZeolite-L

In order to investigate the properties of the Al­(III) complexes adsorbed in a solid matrix, the species were stabilized in the μmZeolite-L template. From the reaction scheme in eq 1, the complex Al­(III)_2_DHQ_2_ (DHQ: QNZ or DHN) should have a net positive charge, allowing an ion exchange process and strong adsorption in μmZeolite-L. Typical confocal fluorescence microscopy images obtained for QNZ/Al­(III) and DHN/Al­(III) are shown in Figure 7. The strong emission toward the center part of the template indicates that the complex with QNZ is entrapped along channel pores of the μmZeolite-L (Figure 7a). On the other hand, the complex with DHN is located preferentially in the poles of the μmZeolite-L (see Figure 7b). Considering that the channel size of μmZeolite-L is approximately 7Å in the entrance diameter and 12.6 Å inside, it allows the diffusion and formation of Al_2_QNZ_2_ with similar diameter, but it precludes formation and accommodation along the channels for Al_2_DHN_2_ because its short diameter is about 13 Å (molecular diameters of the complexes from DFT calculation are reported in SI).

**Figure 7:**
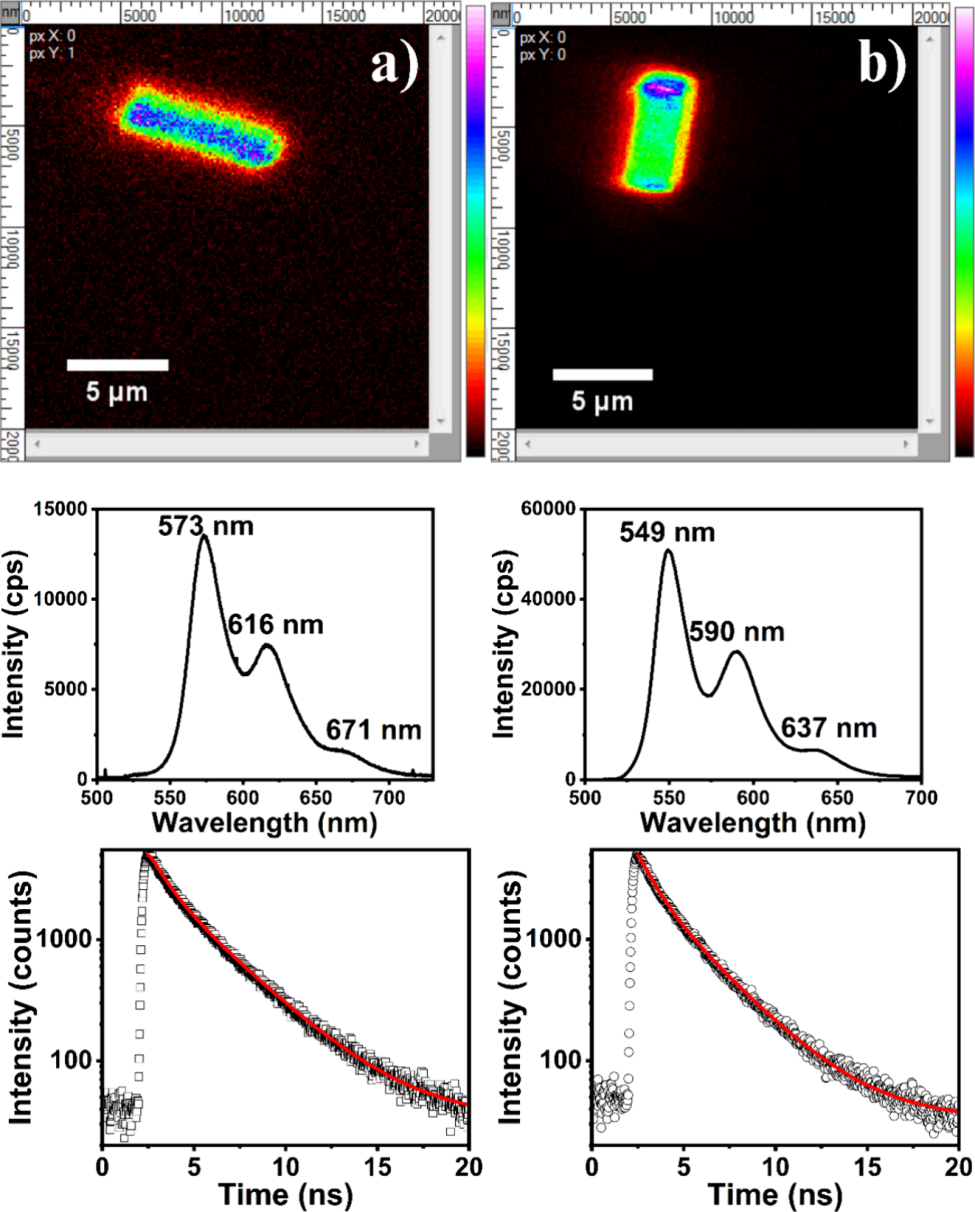
Confocal fluorescence image (top), fluorescence spectra (middle), and fluorescence decay (bottom) of (a) QNZ/Al­(III) and (b) DHN/Al­(III) entrapped in μmZeolite-L.

The fluorescence spectra for both complexes obtained in the zeolite were pretty much the same as in solution, only with a small red shift ascribed to the stabilization of the coordinated structure trapped in the restricted channel of zeolite where slightly different interactions between the complexes can exist due to packing. The fluorescence decay for QNZ/Al­(III) zeolite was nonexponential, with components of 0.92 ns (48%) and 3.05 ns (52%). The DHN/Al­(III) fluorescence decay was also nonexponential with components of 0.89 ns (55%) and 2.77 ns (45%), contrasting with the exponential decay found in ethanol solution in saturated Al concentration (see Figure 5a).

### Quantum Chemical Calculations

To analyze the excitonic coupling in these systems, a simplified model containing two chromophores and two Al^3+^ ions was constructed according to the reaction scheme in eq 1. Each fragment for the electronic transition dipole decomposition includes one chromophore coordinated to one Al^3+^ ion, allowing the study of individual contributions to the overall transition dipole moment. The total molecular charge was set to +3, and the transition selected for the dipole moment calculation corresponded to the HOMO→LUMO excitation, representing the lowest-energy singlet transition responsible for the main optical absorption. The decomposition of the transition dipole of the fragments (red arrows) and the total transition dipole moment (green arrow) can be seen in Figure 8 for the QNZ and DHN Al­(III) systems.

**Figure 8:**
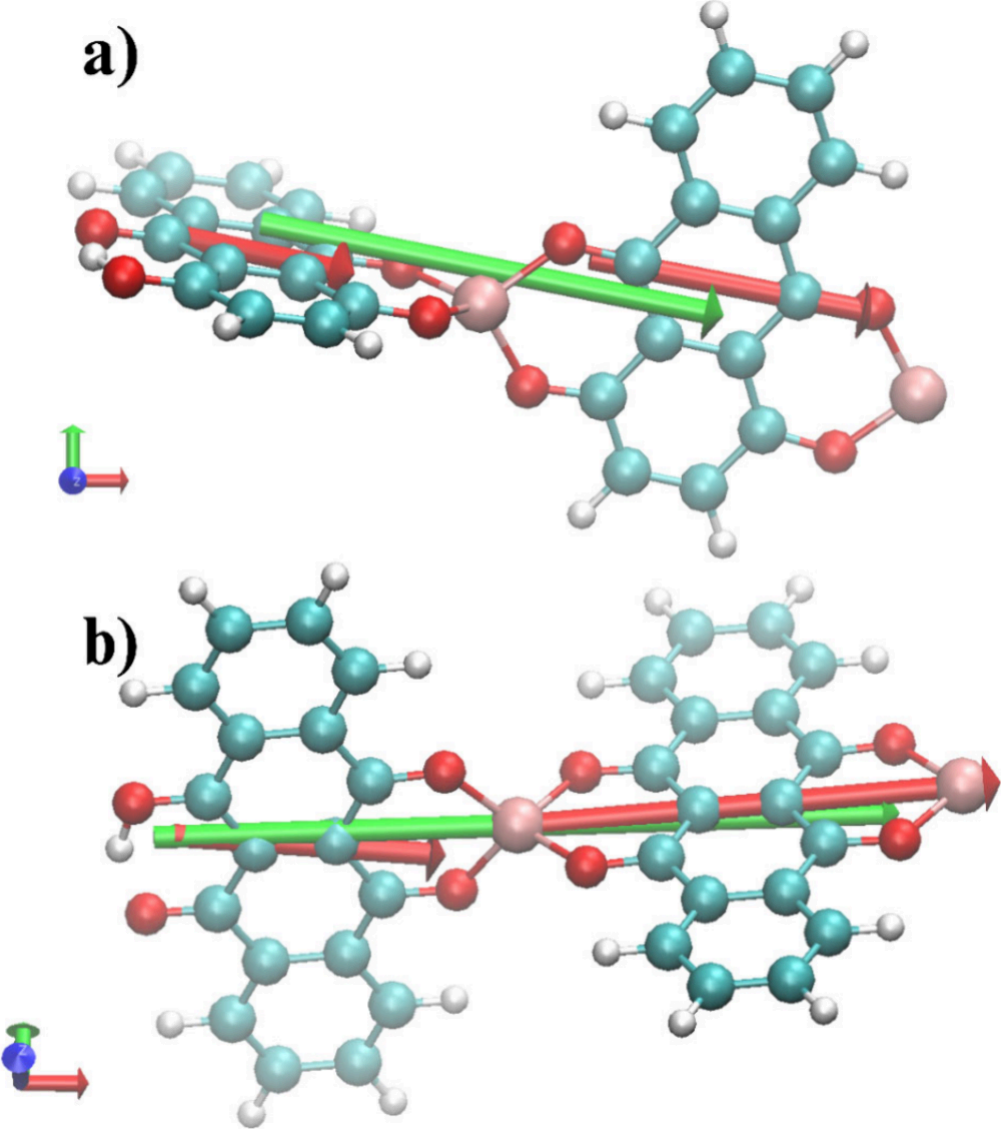
Transition dipole moments of a) Al_2_QNZ_2_ and b) Al_2_DHN_2_. Red arrows indicate the decomposed vectors for each fragment of 1 chromophore and 1 aluminum ion, and the green arrow represents the total transition moment dipole.

To quantify the relative contributions of each chromophore fragment to the total transition dipole moment, a vectorial decomposition was performed. The magnitudes of the fragment transition dipole moments, their angles relative to each other, and their alignments with the total dipole vector were calculated. The cross term in the |V|^2^ decomposition was used to evaluate whether the fragment dipoles were aligned in a parallel or antiparallel manner, thereby enhancing or reducing the total transition dipole moment. Table 2 summarizes these key parameters for both QNZ and DHN, highlighting the fragment alignment, contribution to the total electronic transition dipole, and relative constructive effects indicative of exciton coupling. In QNZ, the two main fragment transition dipoles have magnitudes of 3.19 and 4.74 e a_0_., and they are separated by an angle of 24.0°, with fragment–total angles of 14.4° and 9.6°. This arrangement produces a substantial positive cross term (∼45% of |V|^2^), reflecting strong constructive interference and enhanced total transition dipole magnitude (|V| ≈ 7.77 e a_0_), consistent with J-aggregate–like exciton coupling. In DHN, the fragment dipoles are larger (4.48 and 8.26 e a_0_) and more closely aligned (interfragment angle 11.7°; fragment–total angles 7.6° and 4.1°), resulting in a similar positive cross term (∼45% of |V|^2^) but a significantly larger total dipole magnitude (|V| ≈ 12.68 e a_0_). The near head-to-tail alignment maximizes constructive coupling, leading to a stronger enhancement of the oscillator strength and a more pronounced J-aggregate character. Moreover, the more extended π-system of DHN leads to greater frontier-orbital delocalization, which increases the transition dipole moment and favors geometries with optimal dipole alignment. These electronic-structure features strengthen the excitonic coupling and help explain the stronger J-type behavior of the DHN metal complex. Together with the observed red-shifted and intensified spectral features, these vector alignment patterns support head-to-tail exciton arrangements in both systems, with DHN exhibiting a stronger J-type effect. Moreover, the calculated transitions at 461.1 and 497.8 nm of dimeric models for the Al_2_QNZ_2_ and Al_2_DHN_2_ complexes (see SI) are in consonance with the experimentally observed E_0–0_ corresponding wavelengths at 543 and 565 nm, respectively.

**Table 2:** Key Vectorial Parameters of the Fragment and Total Transition Dipoles for QNZ and DHN^a^

System	|v_1_| [e a_0_]	|v_2_| [e a_0_]	|V| [e a_0_]	θ(v_1_,v_2_) [deg]	θ(v_1_,V) [deg]	θ(v_2_,V) [deg]	Cross term% of |V|^2^	Along V:v_1_ [%]	Along V:v_2_ [%]
Al_2_QNZ_2_	3.19	4.74	7.77	23.97	14.36	9.61	45.12	43.09	56.91
Al_2_DHN_2_	4.48	8.26	12.68	11.70	7.59	4.11	45.08	35.03	64.97

a Magnitudes of fragment and total dipoles (|v_1_|, |v_2_|, |V|), inter-fragment and fragment–total angles, cross term contribution to |V|^2^, and fragment shares along the total dipole vector.

## Conclusions

The spectroscopic results provide evidence for the formation of supramolecular aggregates between dihydroxyquinones and Al­(III) ions. The red-shift in both the absorption and fluorescence, decrease in the Stokes shift, and enhancement of the fluorescence quantum yield corroborate with J-like exciton coupling in the metal-dihydroxyquinone coordination structure. Specifically, the quantum mechanical calculations on dimeric models for the Al_2_QNZ_2_ and Al_2_DHN_2_ complexes reveal a head-to-tail arrangement of the electronic transition dipole moments of the chromophores. This vector alignment, along with the observed spectral emission intensification, is highly characteristic of J-aggregate-like behavior. While both systems exhibit this behavior, DHN demonstrates a more pronounced J-type character due to a larger total dipole magnitude and more closely aligned chromophore fragments. Complexation is also evidenced by the stabilization of the QNZ/Al­(III) and DHN/Al­(III) systems in a μmZeolite-L template, where the fluorescence spectra closely resemble the red-shifted band observed in solution. The fluorescence decay studies further support the formation of chromophore – Al (III) species in solution, as well as in zeolite. However, the increase in fluorescence lifetime observed in both cases contrasts with the behavior found in classical J-aggregate where, due to exciton coupling, the deactivation to the ground state is usually faster when compared to monomer decay. In the present systems, however, the increase in fluorescence lifetime is explained by the decrease in the nonradiative rate constant. These findings elucidate the special light emission character of dihydroxyquinone-Al­(III) complexes, inspiring applications where stable emission is required, such as in OLEDs, fluorescence sensors, and J-aggregates in optical cavities.

## Supplementary Material



## Notes

Solution images, CIE diagram, fluorescence decay analysis, relative emission quantum yield, attenuated total reflection data, molecular geometry and excited state optimization and transition dipole moment vector analysis (PDF)

## References

[1] Trochowski M., Kobielusz M., Pucelik B., Da̧browski J. M., Macyk W. (2023). Dihydroxyanthraquinones as Stable and Cost-Effective TiO2 Photosensitizers
for Environmental and Biomedical Applications. Journal of Photochemistry and Photobiology a Chemistry.

[2] Anouar E. H., Ali Z., Filai I., Abdalla S. (2025). Electronic and Optical Properties
of Functionalized Dihydroxyanthraquinone as Potential Organic Semiconductor
Materials for Solar Cells Applications. J. Phys.
Chem. C.

[3] Yuan, H. ; Cheng, B. ; Lei, J. ; Jiang, L. ; Han, Z. Promoting Photocatalytic CO_2_ Eeduction with a Molecular Copper Purpurin Chromophore. Nat. Commun. 2021, 12 (1). 10.1038/s41467-021-21923-9. PMC7987958 33758178

[4] Mohapatra S. K., Misra M. (2007). Enhanced Photoelectrochemical
Generation of Hydrogen from Water by
2,6-Dihydroxyantraquinone-Functionalized Titanium Dioxide Nanotubes. J. Phys. Chem. C.

[5] Yuan H., Ming M., Yang S., Guo K., Chen B., Jiang L., Han Z. (2024). Molecular Copper-Anthraquinone
Photocatalysts
for Robust Hydrogen Production. J. Am. Chem.
Soc..

[6] Claude J. P., Omberg K. M., Williams D. S., Meyer T. J. (2002). Calculation of Electron
Transfer Rate Constants from Emission Spectra in Re­(I) Chromophore-Quencher
Complexes. J. Phys. Chem. A.

[7] Hu X., Cao Y., Yin X., Zhu L., Chen Y., Wang W., Hu J. (2019). Design and Synthesis
of Various Quinizarin Derivatives as Potential
Anticancer Agents in Acute T Lymphoblastic Leukemia. Bioorg. Med. Chem..

[8] Wang W., Zhang J., Qi W., Su R., He Z., Peng X. (2021). Alizarin and Purpurin from Rubia
tinctorum L. Suppress Insulin Fibrillation
and Reduce the Amyloid-Induced Cytotoxicity. ACS Chem. Neurosci..

[9] Nakayama T., Okumura N., Uno B. (2020). Complementary
Effect of Intra- and
Intermolecular Hydrogen Bonds on Electron Transfer in β-Hydroxy-Anthraquinone
Derivatives. J. Phys. Chem. B.

[10] Gehlen M. H., Simas E. R., Pereira R. V., Sabatini C. A. (2012). Modulation of Dye
Fluorescence by Photoinduced Intramolecular Charge Transfer with Resonance-Assisted
Hydrogen Bond. Reviews in Fluorescence.

[11] Quinti L., Allen N. S., Edge M., Murphy B. P., Perotti A. (2003). A Study of
the Strongly Fluorescent Species Formed by the Interaction of the
Dye 1,4-Dihydroxyanthraquinone (Quinizarin) with Al­(III). J. Photochem. Photobiol., A.

[12] Quinti L., Allen N. S., Edge M., Murphy B. P., Perotti A. (2003). A Study of
the Luminescent Complexes Formed by the Dye 1,4-Dihydroxyanthraquinone
(Quinizarin) and Ga­(III) and In­(III). J. Photochem.
Photobiol., A.

[13] Whan C., Duffin R. N., Burke K. J., Andrews P. C. (2025). Synthesis, Characterization,
and Photophysical Properties of Dimethylgallium­(III) 9,10-Dioxoanthraceneolates. Organometallics..

[14] Wang Y., Luo L., Zhang T., Hu J.-R., Wang H., Bao F., Li C., Sun Y., Li J. (2024). Strategically Engineered Ru­(II) Complexes
with Enhanced ROS Activity Enabling Potent Sonodynamic Effect against
Multidrug-Resistant Biofilms. ACS Appl. Mater.
Interfaces.

[15] Shokrollahi A., Aghaei R. (2014). Spectrophotometric Determination of Trace Amounts of
Al^3+^ Ion in Water Samples after Cloud Point Extraction
Using Quinizarin as a Complexing Agent. Environmental
Monitoring and Assessment.

[16] Shahzadi K., Mansha A., Asim S. (2025). The Fluorescence
Sensing Capability
of 1,4-dihydroxyanthraquinone Towards Metal Ions and Imaging Cells. Journal of Fluorescence..

[17] Coble H.D., Holtzclaw H. F. (1974). Chelate Polymers of Copper­(II) with
Various Dihydroxyquinoid
Ligands. Journal of Inorganic and Nuclear Chemistry.

[18] Xu Y., Zhao Y., Zhang J., Wang X., Gao S., Wang Z., Qiao W., Wang Z. Y. (2023). Tuning of Molecular
Aggregation and Photoresponse of Narrow-band Organic Photodetectors. ACS Applied Electronic Materials.

[19] Takahashi M., Sakai K.-i., Sambe K., Akutagawa T. (2022). Supramolecular
Complexation and Collective Optical Properties Induced by Linking
Two Methyl Salicylates via a σ-Bridge. J. Phys. Chem. B.

[20] Rajput D., Sanyam, Rawat G., Sorout P., Kanvah S., Mondal A. (2024). From Molecule to Aggregate:
Understanding AIE through Multiscale Experimental and Computational
Techniques. J. Phys. Chem. B.

[21] Portwich, F. L. ; Carstensen, Y. ; Dasgupta, A. ; Kupfer, S. ; Wyrwa, R. ; Görls, H. ; Eggeling, C. ; Dietzek, B. ; Gräfe, S. ; Wächtler, M. ; Kretschmer, R. A Highly Fluorescent Dinuclear Aluminium Complex with Near-Unity Quantum Yield**. Angew. Chem., Int. Ed. 2022, 61(17). 10.1002/anie.202117499. PMC9313782 35107199

[22] Klimenko I. V., Astakhova T. Y., Timokhina E. N., Lobanov A. V. (2023). Molecular Aggregation
of Aluminum Phthalocyanine Chloride in Organic and Water-Organic Media. Journal of Biomedical Photonics & Engineering.

[23] Khan T., Vaidya S., Mhatre D. S., Datta A. (2016). The Prospect of Salophen
in Fluorescence Lifetime Sensing of Al3. Journal
of Physical Chemistry. B.

[24] Guo Y., Chen K., Hu Z., Lei Y., Liu X., Liu M., Cai Z., Xiao J., Wu H., Huang X. (2022). Metal Ions
as the Third Component Coordinate with the Guest to Stereoscopically
Enhance the Phosphorescence Properties of Doped Materials. J. Phys. Chem. Lett..

[25] Tohora N., Ahamed S., Sahoo R., Mahato M., Sultana T., Lama S., Maiti A., Das S. K. (2024). Solid-state brightness
and Al3+ ions-triggered flower-shaped nano-luminogen for cascade detection
of Al3+ and PO43- ions. Opt. Mater..

[26] Hestand N. J., Spano F. C. (2018). Expanded Theory
of H- and J-Molecular Aggregates: The
Effects of Vibronic Coupling and Intermolecular Charge Transfer. Chem. Rev..

[27] Nunzi F., Fantacci S., De Angelis F., Sgamellotti A., Cariati E., Ugo R., Macchi P. (2008). Theoretical
Investigations
of the Effects of J-Aggregation on the Linear and Nonlinear Optical
Properties of E-4-(4-Dimethylaminostyryl)-1-methylpyridinium [DAMS+]. J. Phys. Chem. C.

[28] Tamura H., Hamada I., Shang H., Oniwa K., Akhtaruzzaman M., Jin T., Asao N., Yamamoto Y., Kanagasekaran T., Shimotani H., Ikeda S., Tanigaki K. (2013). Theoretical Analysis
on the Optoelectronic Properties of Single Crystals of Thiophene-furan-phenylene
Co-Oligomers: Efficient Photoluminescence due to Molecular Bending. J. Phys. Chem. C.

[29] Kasha M. (1963). Energy Transfer
Mechanisms and the Molecular Exciton Model for Molecular Aggregates. Radiat. Res..

[30] Hestand N. J., Spano F. C. (2017). Molecular Aggregate Photophysics beyond the Kasha Model:
Novel Design Principles for Organic Materials. Acc. Chem. Res..

[31] Xu Y., Zhao Y., Zhu H., Li Y., Gong H., Lv B., Luo N., Zhao B., Qiao W., Wang Z. Y. (2024). The Aggregation
Units of J-Aggregates: Transitioning from Monomers to Hydrogen-Bonded
Dimers. J. Phys. Chem. Lett..

[32] Shabbir A., Shahzad S. A., Alzahrani A. Y. A., Khan Z. A., Yar M., Rauf W. (2025). A Multimode Fluorescent
Sensor for Sequential Detection of Cu^2+^ and Cysteine as
well as a pH Sensor with Real Sample Applications:
Extensive Experimental and DFT Studies. Spectrochimica
Acta Part A: Molecular and Biomolecular Spectroscopy.

[33] He Z., Gao Y., Huang Z., Zhan M., Tian S., Fang F., Zhao D., Li Z., Meng F., Tang B. Z., Luo L. (2025). Tuning the Near-Infrared
J-Aggregate of a Multicationic Photosensitizer
through Molecular Coassembly for Symbiotic Photothermal Therapy and
Chemotherapy. ACS Nano.

[34] Xu Y., Meng X., Zhao Y., Jia M., Zhu H., Song J., Su Y., Qiao W., Qi J., Wang Z. Y. (2024). Pyrrolopyrrole Cyanine J-Aggregate Nanoparticles with
High Near-Infrared Fluorescence Brightness and Photothermal Performance
for Efficient Phototheranostics. ACS Applied
Materials & Interfaces..

[35] Chen T., Lin X., Li P., Liu W., Jiang L., Huang L., Li Z., Zhu R., Chen Z.-L., Zeng R., Liu M., Chen W. (2026). J-Aggregate Fluorescence Probe Constructed Based on AIE Units for
Analysis of Zn^2+^ and Cu^2+^ Ions in Food and Biological
Systems. Spectrochimica Acta Part A: Molecular
and Biomolecular Spectroscopy.

[36] Lencione D., Gehlen M. H., Trujillo L. N., Leitao F., Albuquerque R. Q. (2016). The Spatial
Distribution of the Photostability of Thionine in Zeolite L Nanochannels
Investigated by Photobleaching Lifetime Imaging Microscopy. Photochemical & Photobiological Sciences.

[37] Casey K. G., Quitevis E. L. (1988). Effect of solvent
polarity on nonradiative processes
in xanthene dyes: Rhodamine B in normal alcohols. J. Phys. Chem..

[38] Reis I. F., Foltran L. S., Lauer M. H., Gehlen M. H., Drekener R. L., Correia C. R. D. (2021). Reactive Phenanthrene
Derivatives as Markers of Amino
Groups in Fluorescence Microscopy of Surface Modified Micro-Zeolite
L. J. Fluoresc..

[39] Neese, F. Software Update: The ORCA Program System—Version 6.0. WIREs Computational Molecular Science 2025, 15 (2).10.1002/wcms.70019.

[40] Weigend F., Ahlrichs R. (2005). Balanced Basis Sets of Split Valence, Triple Zeta Valence,
and Quadruple Zeta Valence Quality for H to Rn: Design and Assessment
of Accuracy. Phys. Chem. Chem. Phys..

[41] Weigend F. (2006). Accurate Coulomb-fitting
basis sets for H to Rn. Phys. Chem. Chem. Phys..

[42] Becke A. D. (1993). Density-Functional
Thermochemistry. III. The Role of Exact Exchange. J. Chem. Phys..

[43] Stephens P. J., Devlin F. J., Chabalowski C. F., Frisch M. J. (1994). Ab Initio Calculation
of Vibrational Absorption and Circular Dichroism Spectra Using Density
Functional Force Fields. J. Phys. Chem..

[44] Yanai T., Tew D. P., Handy N. C. (2004). A New Hybrid Exchange-Correlation
Functional Using the Coulomb-Attenuating Method (CAM-B3LYP). Chem. Phys. Lett..

[45] Lu T., Chen F. (2012). Multiwfn: A Multifunctional
Wavefunction Analyzer. J. Comput. Chem..

[46] Humphrey W., Dalke A., Schulten K. (1996). VMD: Visual
Molecular Dynamics. J. Mol. Graphics.

[47] Barotov, U. , Thanippuli Arachchi, D. H. , Klein, M. D. , Zhang, J. , Sverko, T. , Bawendi, M. G. Near-Unity Superradiant Emission from Delocalized Frenkel Excitons in a Two-Dimensional Supramolecular Assembly. Advanced Optical Materials 2023, 11 (2). 10.1002/adom.202201471. PMC9957265 36846517

