# Supplementary material for: Induction of J Aggregate-like Optical Transitions in Dihydroxyquinone by Coordination with Al(III)
Source: J Phys Chem A. 2026 Jan 29;130(6):1275–81. doi: 10.1021/acs.jpca.5c06958 (PMC12908156; doi:10.1021/acs.jpca.5c06958)
Supplement: Supplementary file 1 [file jp5c06958_si_001.pdf]

The Induction of J Aggregate-like Optical Transitions in Dihydroxyquinone by Coordination with Al(III)

José Roberto Granado Neto, Antonio Gustavo Sampaio de Oliveira-Filho, Marcelo Henrique Gehlen*

Institute of Chemistry of São Carlos – University of São Paulo

* Corresponding author (marcelog@iqsc.usp.br)

Supplementary Information

The CIE chromaticity diagrams were generated using the Origin 2022b Chromaticity Diagram App. The chromaticity coordinates were calculated directly from the spectroscopic datasets. For the absorption measurements, the spectra were first converted into the corresponding reflected spectra under the standard D65 daylight illuminant, as required for accurate colorimetric evaluation.

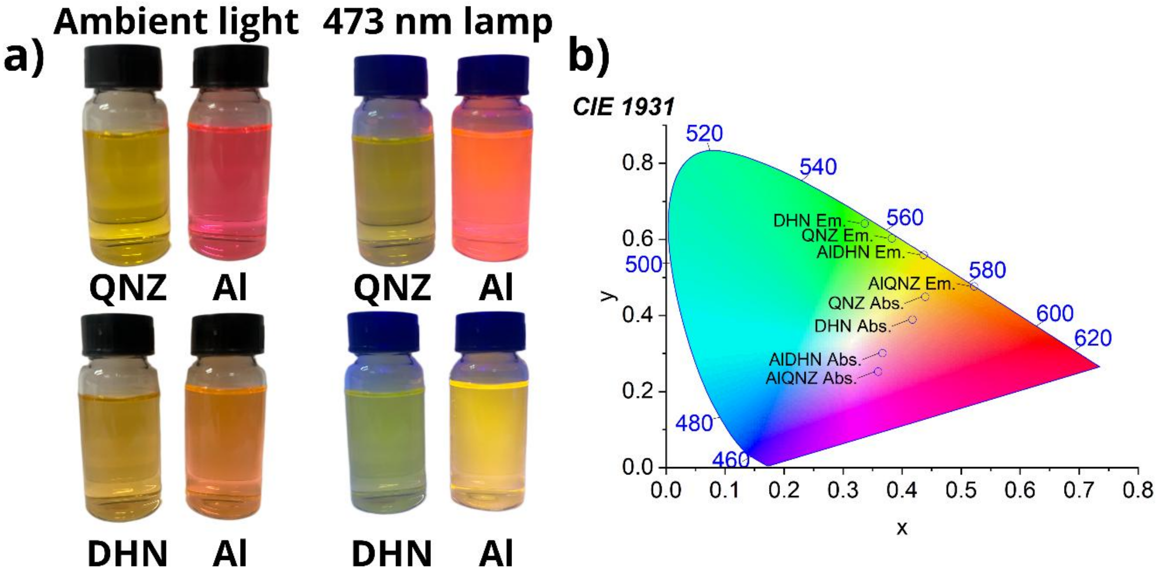

Figure S1. a) Picture of the ethanolic solutions of QNZ, QNZ/Al(III), DHN and DHN/Al(III), under ambient light and incidence of blue light (473 nm). b) CIE diagram of the solutions using spectroscopy data. Absorption data were converted to reflected spectrum under defined illuminant (sunlight D65).

Table S1. Bi-exponential fluorescence decay times (τ_1 and τ_2), average (τ_{avg}), and normalized pre-exponential factors (b_1 and b_2) for QNZ solution at different molar equivalents of Al(III).

System	Molar equivalent Al(III)	τ_1 (ns)	τ_2 (ns)	b_1	b_2	τ_{avg} (ns)
QNZ	0.0	0.60	0.99	0.92	0.08	0.65
	0.2	0.61	0.78	0.89	0.11	0.63
	1.0	0.61	1.21	0.90	0.10	0.71
	2.0	0.65	1.62	0.29	0.71	1.49
	4.0	1.09	2.88	0.24	0.76	2.69
	10	1.20	3.37	0.66	0.34	2.48
	20	1.24	3.34	0.50	0.50	2.77

Table S2. Bi-exponential fluorescence decay times (τ_1 and τ_2), average (τ_{avg}) and normalized pre-exponential factors (b_1 and b_2) for DHN solution and different molar equivalents of Al(III).

System	Molar equivalent Al(III)	τ_1 (ns)	τ_2 (ns)	b_1	b_2	τ_{avg} (ns)
DHN	0.0	1.35	1.61	0.19	0.81	1.57
	0.2	0.83	1.78	0.14	0.86	1.71
	1.0	1.30	2.12	0.45	0.55	1.85
	2.0	1.29	2.23	0.43	0.57	1.95
	4.0	2.46	3.02	0.82	0.18	2.58
	10	2.61	0.00	1.00	0.00	2.61
	20	2.61	0.00	1.00	0.00	2.61

Table S3. Bi-exponential fluorescence decay times (τ_1 and τ_2), average (τ_{avg}) and normalized pre-exponential factors (b_1 and b_2) for µZeolite-L labeled with QNZ/Al and DHN/Al.

System	τ_1 (ns)	τ_2 (ns)	b_1	b_2	τ_{avg} (ns)
µZeo/QNZ/Al	0.92	3.05	0.48	0.52	2.59
µZeo/DHN/Al	0.89	2.77	0.55	0.45	2.24

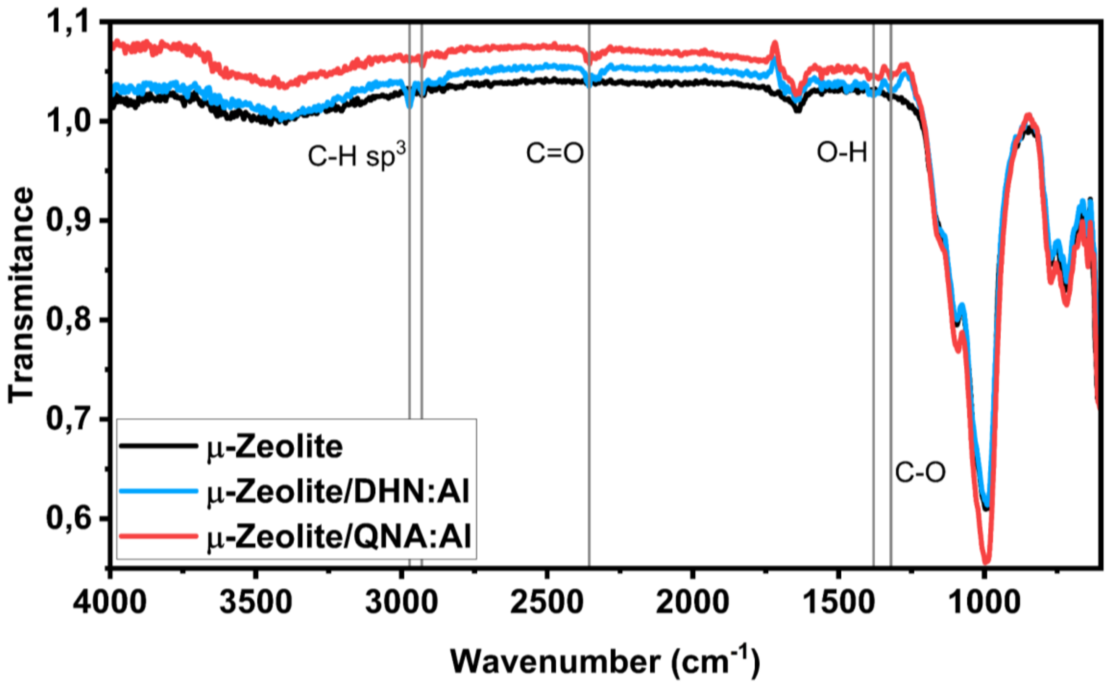

Figure S2. Attenuated total reflection (ATR) spectra of the pure µmZeolite-L, and its labeled form by QNZ/Al(III) and DHN/Al(III), showing principal infrared changes.

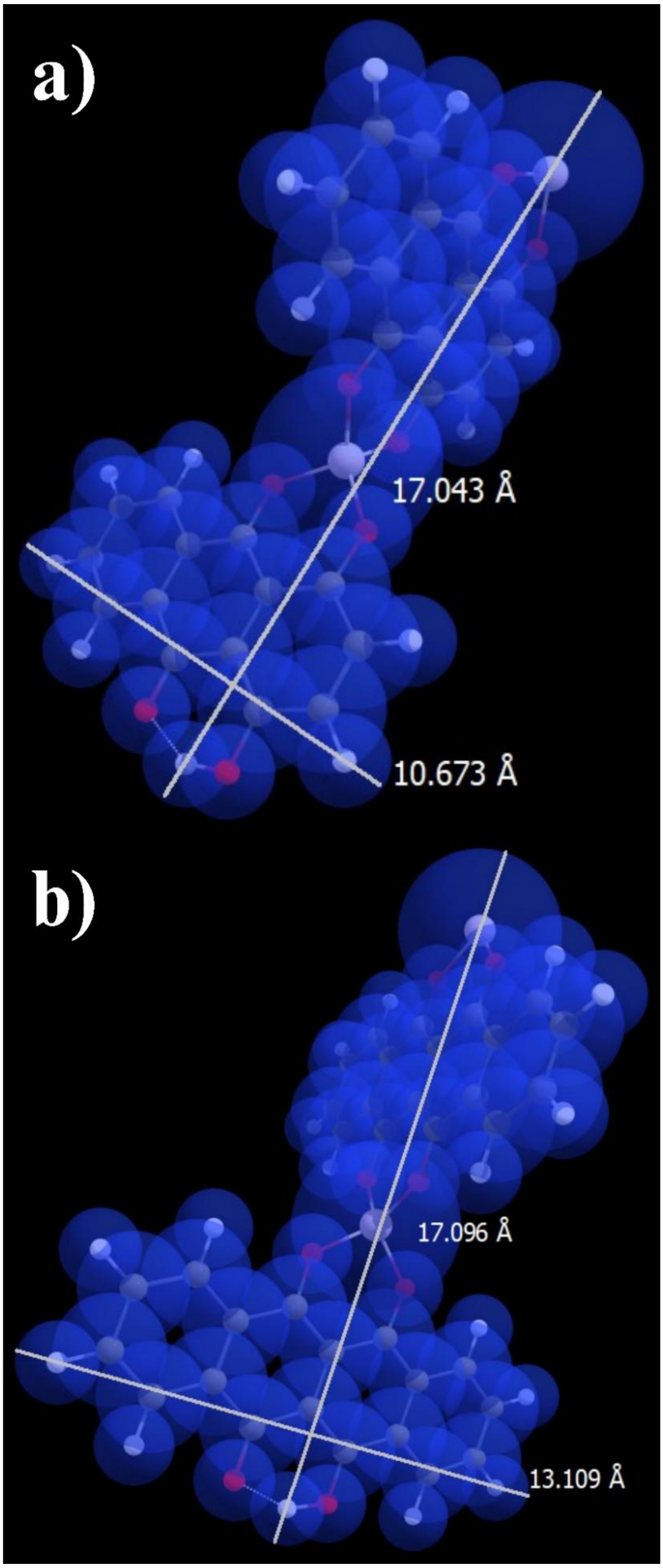

Figure S3. Optimized geometry of (a) Al_2QNZ_2 and (b) Al_2DHN_2 with Van Der Walls spheres in blue, showing its molecular diameters in Angstroms.

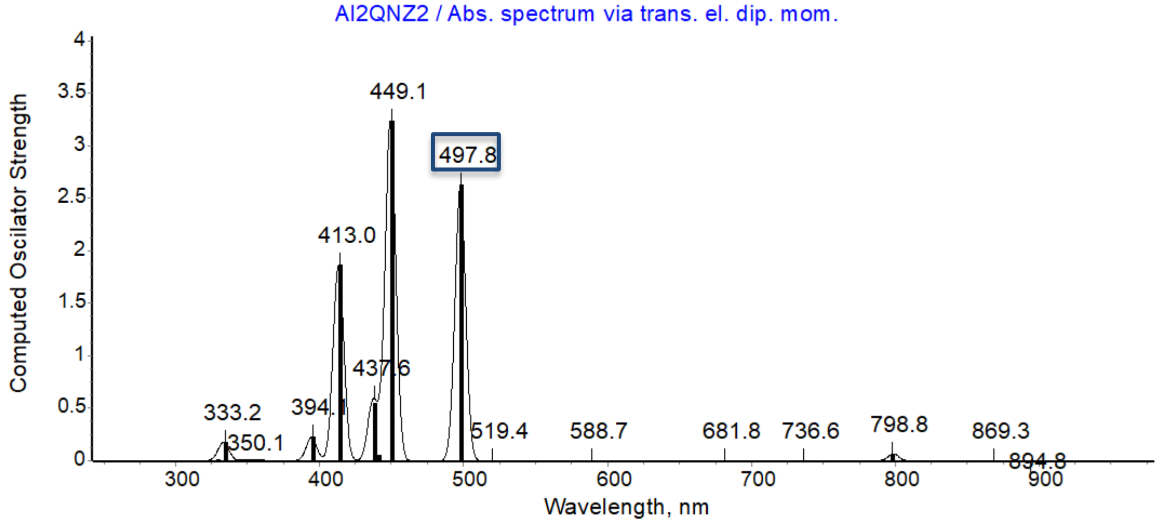

Figure S4. TDDFT absorption spectra via transition electron dipole moment for Al_2QNZ_2. The 497.8 nm peak was used for transition dipole decomposition.

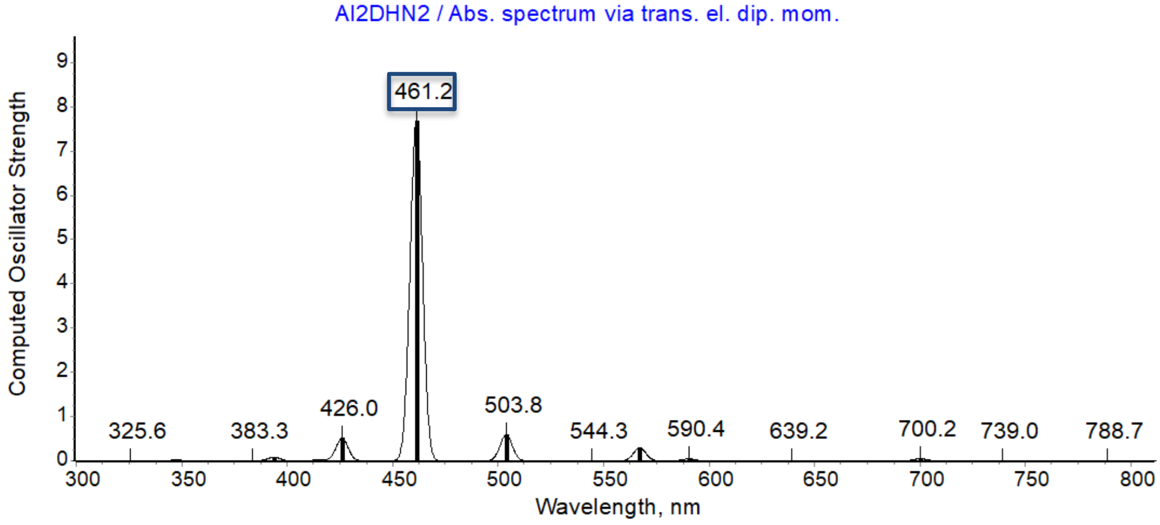

Figure S5. TDDFT absorption spectra via transition electron dipole moment for Al_2DHN_2. The 461.2 nm peak was used for transition dipole decomposition.

Transition Dipole Vector Decomposition

The transition dipole moment of the total system, V, was expressed as the sum of the fragment contributions v_1 and v_2:

$$V = v_1 + v_2$$

The squared magnitude of the total vector was then decomposed as:

$$|V|^2 = |v_1|^2 + |v_2|^2 + 2\, v_1 \cdot v_2$$

where the cross term represents the parallel or antiparallel alignment the two fragment of transition dipoles.

Vector Magnitudes

The magnitude of each vector was calculated as the Euclidean norm:

$$|v_i| = \sqrt{v_{ix}^2 + v_{iy}^2 + v_{iz}^2}, \quad i = 1,2$$

and for the total dipole:

$$|V| = \sqrt{V_x^2 + V_y^2 + V_z^2}$$

Angular Relations

The angle between two vectors was obtained using the scalar product:

$$\theta(v_i, v_j) = \cos^{-1}\left[\frac{(v_i \cdot v_j)}{(|v_i||v_j|)}\right]$$

This expression was applied to determine the inter-fragment angle $\theta(v_1, v_2)$ as well as the angle of each fragment with respect to the total dipole vector $\theta(v_i, V)$.

Projection onto the Total Dipole

The relative contribution of each fragment along the direction of the total transition dipole was evaluated by projecting each fragment vector onto V:

$$p_i = \frac{(v_i \cdot V)}{|V|^2}$$

which represents the fractional share of the total transition dipole carried by fragment i.

Cross Term Contribution

Finally, the percentage contribution of the cross term to the total squared transition dipole was calculated as:

$$\%cross\ term = \left[\frac{2(v_1 \cdot v_2)}{|V|^2}\right] \times 100\%$$

This quantifies the extent of parallel or antiparallel alignment between the two fragment transition dipoles.
